# Urinary Exosomal microRNAs as a Novel Approach to Study People with Multiple Sclerosis and Severe Gait Disability: A Preliminary Observation

**DOI:** 10.3390/ncrna12030016

**Published:** 2026-05-08

**Authors:** Silvia Grassilli, Andrea Baroni, Marina Pierantoni, Federica Brugnoli, Nicola Lamberti, Sofia Straudi, Fabio Manfredini, Valeria Bertagnolo

**Affiliations:** 1 Department of Environmental Sciences and Prevention and LTTA Centre, University of Ferrara, 44121 Ferrara, Italy or grsslv@unife.it; 2 Department of Neuroscience and Rehabilitation, University of Ferrara, 44121 Ferrara, Italy; brnndr3@unife.it (A.B.); lmbncl@unife.it (N.L.); strsfo@unife.it (S.S.); fabio.manfredini@unife.it (F.M.); 3 Unit of Physical and Rehabilitation Medicine, University Hospital of Ferrara, 44124 Ferrara, Italy; 4 Department of Translational Medicine, University of Ferrara, 44121 Ferrara, Italy; marina.pierantoni@unife.it; 5 Program of Vascular Rehabilitation and Exercise Medicine, University Hospital of Ferrara, 44124 Ferrara, Italy

**Keywords:** urinary exosomes, miRNAs, multiple sclerosis, non-invasive biomarkers, rehabilitation

## Abstract

**Background:** MiRNAs within extracellular vesicles can encompass body barriers, reflecting stage, progression, and response to treatments of various diseases, including multiple sclerosis (MS)—a chronic immune-mediated disease of the central nervous system that causes progressive disability, with highly variable clinical courses. In this context, urinary exosomal miRNAs could be an appealing source of biomarkers, thanks to their non-invasive and easily repeatable collection. **Methods:** In this exploratory investigation, we tried to assess if profiling urinary exosomal miRNAs could reveal subtle differences within an apparently homogeneous MS population. The study involved 24 patients with primary or secondary progressive MS, whose urinary exosomes (UEs) were subjected to evaluation of a panel of 87 miRNAs variously correlated with neuroinflammation, cardiovascular functions, and/or involved in MS. **Results:** We revealed that the examined miRNAs were heterogeneously expressed across the patients, reflecting, as expected, their gender and/or hormonal status. Two miRNAs discriminated against primary or secondary progressive MS, and a panel of 14 commonly upmodulated miRNAs identified patients with longer disease duration and a greater degree of disability. **Conclusions:** Even if preliminary, these data represent the first relationship between UEs and MS features in humans and suggest that urine could constitute a non-invasive source of exosomal miRNAs, which could prove useful in complementing conventional monitoring to provide a more personalized management of MS patients.

## 1. Introduction

Multiple sclerosis (MS) is an immune-mediated disease of the central nervous system (CNS) characterized by demyelination associated with compartmentalized inflammation, mitochondrial dysfunction and altered immune responses, constituting one of the leading causes of permanent neurological disability in young adults, with an estimated 2.9 million people affected worldwide in 2023 [1,2].

The clinical course of MS is highly variable, allowing clinicians to classify the disease as relapsing-remitting (RRMS) or progressive. Progressive forms of MS include primary progressive MS (PPMS), affecting around 15% of MS patients, which shows continuous worsening from clinical onset of disease, typically without relapses but with possible periods of plateau. At variance, secondary progressive MS (SPMS) is characterized by steady progression, sometimes with acute relapses, and accumulation of disability affecting motor, autonomic and cognitive functions. In this context, an early and accurate diagnosis, as well as the monitoring of disease progression, require a comprehensive evaluation of clinical history, neuroimaging, laboratory analysis, and neurophysiological assessment [3,4,5,6].

For the monitoring of various diseases, including MS, growing attention is being directed to the evaluation of biomarkers in body fluids, mainly in blood and cerebrospinal fluid (CSF). In the context of MS, the evaluation of panels, rather than single molecules, helps clinicians to better distinguish MS from other neurological disorders and provide more accurate predictions of relapse risk and disability progression [7,8]. From a biomolecular perspective, the identification of reliable biomarkers of MS progression and response to rehabilitation treatment remains a critical challenge [5,7]. In recent years, increasing attention has been directed toward microRNAs (miRNAs), small non-coding RNAs involved in the post-transcriptional regulation of gene expression, that can be released into extracellular environments where they can reach body fluid such as plasma, saliva, and urine [9,10]. Extracellular miRNAs can be encapsulated in extracellular vesicles (EVs), particularly exosomes, which are secreted by cells and implicated in intercellular communication. MiRNA packaging into exosomes is a selective process, reflecting the physiological or pathological state of the cell of origin, while the exosomal membrane composition influences their uptake by recipient cells [11,12]. Exosomes can cross the blood–brain barrier, making them especially appealing as non-invasive indicators of CNS pathologies [13], and exosomal microRNAs from blood/serum and cerebrospinal fluid (CSF) can reflect stage, progression, and response to treatments of MS patients [14,15,16,17].

Exosomes can also cross the filtration barrier, and urinary exosomes have been shown to reflect diseases not specifically correlated to urogenital apparatus [18,19]. Given the intrinsic vulnerability of individuals with MS, urine has therefore emerged as an attractive, non-invasive source of potential biomarkers, with evidence of inflammatory and neurodegenerative signal molecules relevant to MS [18,19]. As urinary miRNAs could constitute particularly appealing biomarkers, this manuscript aimed to assess if the profiling of urinary exosomal miRNAs of MS patients may offer new insights into disease heterogeneity and/or provide useful information to better monitor the disease stage, the response to therapies and, ultimately, how pathology influences overall patient health.

## 2. Results

### 2.1. Identification of Urinary Exosomal miRNAs in MS Patients

**Figure 1:**
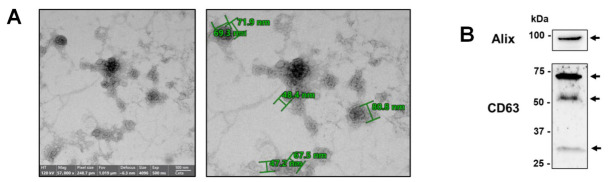
Characterization of purified exosomes. (**A**) Representative transmission electron microscopy images showing purified EVs (36,000× magnification). Specific vesicle sizes are indicated in the image on the right (73,000× magnification). (**B**) Western blot analysis of the transmembrane exosomal markers CD63 and Alix in vesicles purified from the urine of MS patients. The heavily (60–75 kDa), moderately (45–55 kDa), and non-glycosylated (25–30 kDa) CD63 forms are indicated by arrows.

In this study, EVs were extracted from the urine samples of 24 MS patients using the ultracentrifugation procedure. Transmission electron microscopy (TEM) established a cup-shaped structure and a size range of 30–150 nm of purified vesicles, compatible with exosomes (Figure 1A). Western blot analysis of the exosomal marker CD63 revealed the predominant presence of moderately and heavily glycosylated and faint non-glycosylated forms of tetraspanin (Figure 1B) [20]. The abundance of the “endosomal sorting complexes required for transport” (ESCRT) protein Alix [21] confirmed the enrichment in exosomes of the EVs purified from urine samples.

**Figure 2:**
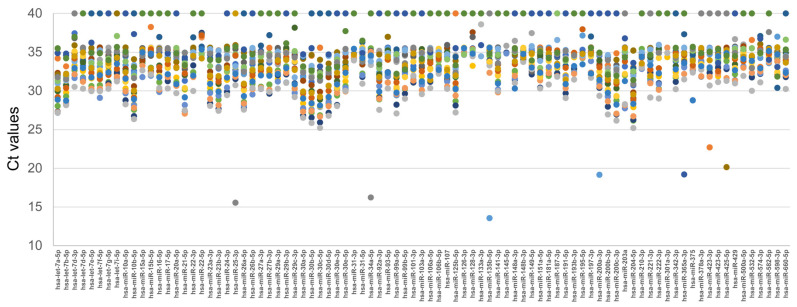
Ct values of the 87 miRNAs analyzed in the UEs of 24 MS patients. The *X*-axis shows the different miRNAs analyzed, while the *Y*-axis displays the corresponding Ct values in each patient, indicated with colored circles. Undetermined samples were placed at Ct 40.

Purified urinary exosomes (UEs) were subjected to RNA extraction and qPCR to explore the differential expression profile of 87 urinary exosomal miRNAs known to be variably associated with neurogenesis, neuronal activity and/or cardiovascular function, and/or found in CSF exosomes. The qPCR analysis also included five RNA spike-ins not endogenously expressed in human samples, added in a fixed amount to each sample during RNA isolation and used to monitor RNA extraction efficiency and to normalize technical variability across samples. As shown in Figure 2, all miRNAs were valuable and analyzed in the MS cohort, with most Ct values between 30 and 36, comparable to those reported in the literature for exosomal miRNAs [22,23]. Some miRNAs were not detected in specific patients and were set to 40 Ct. Ct values ≤ 24 were considered contaminations of non-exosomal miRNAs and were not considered in the subsequent analyses.

**Figure 3:**
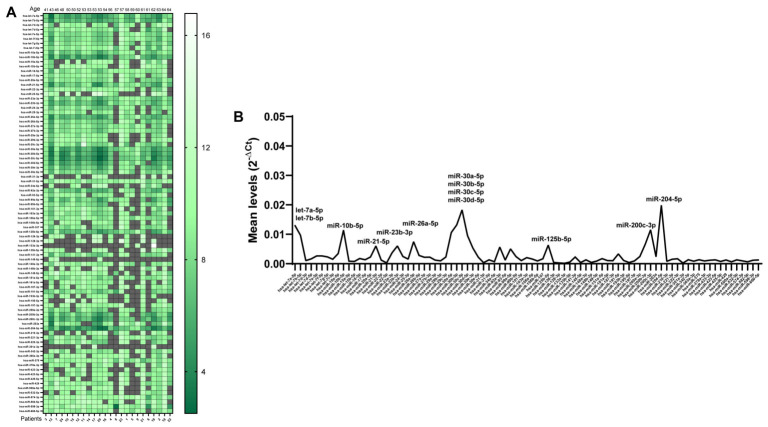
Levels of the 87 urinary exosomal miRNAs in MS patients ordered by age. In (**A**), miRNA levels (ΔCt) were obtained by comparison with a spike-in miRNA included in the analysis. MiRNA intensities are displayed as a growing green color scale. ΔCt undetermined values, due to Ct = 40 and Ct ≤ 24, are shown in gray. In (**B**), a line graph representing the mean expression of each miRNA is shown, expressed as 2^−ΔCt^. MiRNAs present at higher levels are indicated.

To assess the levels of all examined miRNAs in the UEs of the MS patients, their ΔCt values were calculated with respect to an internal spike-in control. The control was selected because out of the five included in the analysis, it showed levels closer to those of the patients’ miRNAs (Figure 3A). Considering that miRNAs whose Ct level was 40 or ≤24 were not considered (dark in the figure), we observed that 68 of the 87 miRNAs were revealed in at least two-thirds of the samples, confirming that the urinary exosomal miRNAs panel we selected was evaluable in our UE samples. A total of 11 miRNAs were present in all patients (miR-let-7a-5p, miR-let-7b-5p, miR-10b-5p, miR-23a-3p, miR23b-3p, miR-30a-5p, miR-31-5p, miR-204-5p, miR-375, miR-574-3p and miR-598-3p), while three miRNAs (miR-133a-3p, miR-145-5p, and miR-301a-3p) were revealed in the urinary exosomes of only three patients. Despite the high variability among patients, the miRNAs showing the higher average levels in purified urinary exosomes were let-7a-5p, let-7b-5p, miR-10b-5p, miR-21-5p, miR-23b-3p, miR-26a-5p, miR-30a-5p, miR-30b-5p, miR-30c-5p, miR-30d-5, miR-125b-5p, miR-200c-3p and miR-204-5p (Figure 3B).

### 2.2. Correlation Between Levels of Urinary Exosome miRNAs and Age/Gender of MS Patients

**Figure 4:**
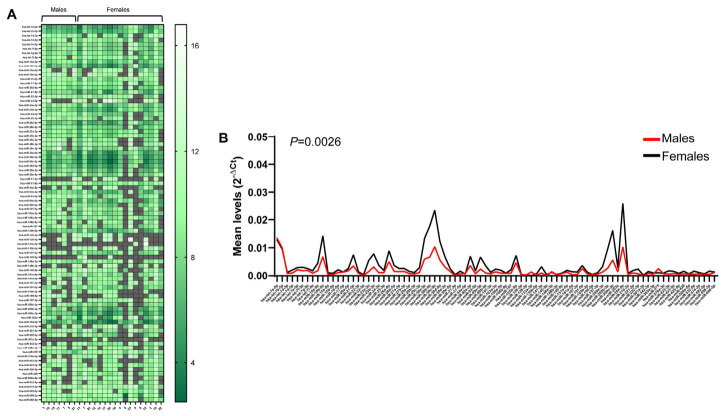
Levels of urinary exosomal miRNAs in MS patients clustered in male and female groups in order of age. In (**A**), ΔCt levels were obtained by comparing each miRNA with a spike-in control RNA. MiRNA intensities are displayed as a growing green color scale. Missing values are shown in gray. In (**B**), line graphs represent the mean expression of each miRNA in the male and female groups, expressed as 2^−ΔCt^.

To assess the distribution of miRNA levels in the studied MS cohort, we first examined the urinary exosomal miRNA profiles in male (*n* = 7) and female (*n* = 17) groups. As shown in Figure 4A, where MS patients were clustered and miRNAs levels were normalized with the internal control (spike-in), males showed a higher number of undetected miRNAs than females, with 12 miRNAs in males and nine miRNAs in females revealed in less than half of the respective patient group. Furthermore, two miRNAs (miR-145-5p and miR-301a-3p) were absent in all seven male patients and, even if some miRNAs were expressed in only a few patients, no miRNA was absent in all 17 female patients (Figure 4A). Accordingly, while the most abundant miRNAs were the same in the two patient groups, an overall higher miRNA level in females than in males (*p* = 0.0026) was revealed (Figure 4B). Of note, both male and female groups showed similar levels of the let 7 family members, essential for the control of development, cell proliferation and tissue differentiation in humans [24], constituting a sort of internal control for the procedure used, and suggesting the need to explore the rationale at the basis of the gender-related differences revealed in the UEs of our MS cohort.

**Figure 5:**
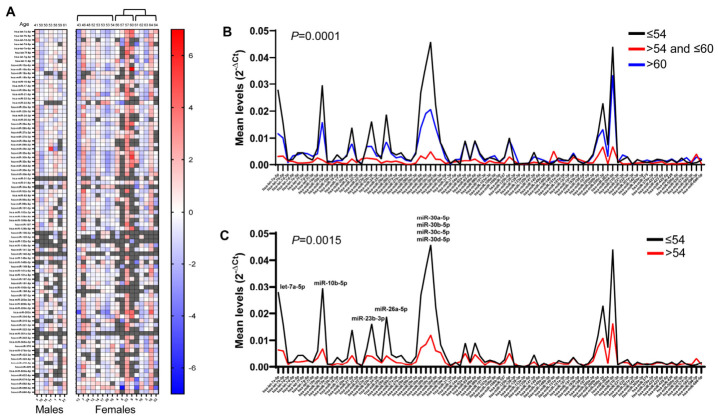
Levels of urinary exosomal miRNAs in female MS patients clustered by age. In (**A**), levels (ΔCt) were obtained by comparing each miRNA level with its global mean in the male and female groups. MiRNA intensities are displayed as colors ranging from blue (higher than average) to red (lower than average). Missing values are shown in gray. In (**B**,**C**), line graphs represent the mean expression of each miRNA in the female groups clustered by age, expressed as 2^−ΔCt^. Significantly different miRNA levels are indicated.

When the CT value of each miRNA in the two groups was compared with its average, we revealed that males show a homogeneous pattern, as miRNAs levels are close to their mean values (Figure 5A), while females show more variable miRNA levels (from dark red to dark blue). Since the heat maps showed the presence of a group of female patients aged between 56 and 60 with a high number of miRNAs not detected or with levels lower than the average, a first analysis was performed comparing the miRNA levels of female patients clustered by age in three groups: up to 54 years, between 55 and 60 years, and over 60 years of age. As shown in Figure 5B, female patients between 55 and 60 years old showed a significantly (*p* < 0.0001) lower global mRNA level than patients ≤54 and >60. On the other hand, patients ≤54 showed the highest miRNA levels, with no significant difference with respect to patients aged >60 years, showing intermediate miRNA levels. We then evaluated the average level of each miRNA using 54 years as a cut-off age, revealing that younger female patients showed an overall higher level of the examined miRNAs than older patients (*p* = 0.0015), with some miRNAs differentially expressed. The significantly higher level of miRNAs in younger females included miR-10b-5p, miR-23b-3p, miR-26a-5p, miR-30a-5p, miR-30b-5p, miR-30c-5p, and miR-30d-5p (Figure 5C).

### 2.3. Correlation of Exosomal Urinary miRNA Levels with MS-Related Parameters

**Figure 6:**
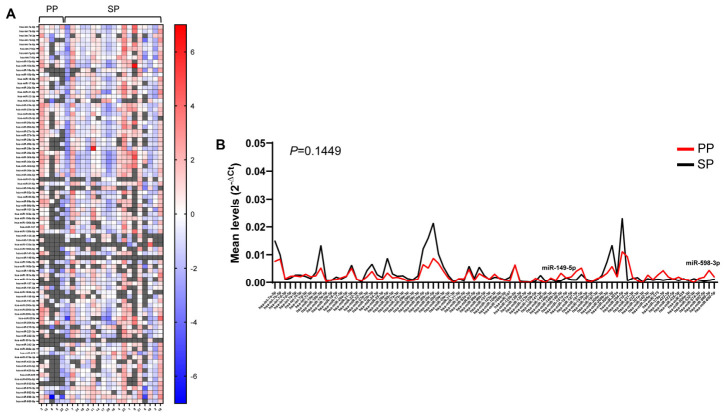
Correlation of urinary exosomal miRNAs with PPMS and SPMS. In (**A**), heat maps show ΔCt levels of urinary exosomal miRNAs in PP and SP patients, using the global mean level of each miRNA as a reference. Missing values are shown in gray. In (**B**), line graphs represent the analysis of mean levels of each miRNA in the two populations (PP and SP), expressed as 2^−ΔCt^. Significantly different miRNA levels are indicated.

To investigate whether differences in urinary exosomal miRNA levels between patients are associated with specific clinicopathological parameters, we first analyzed their correlation with primary (*n* = 5) or secondary progressive MS (*n* = 19). As shown in Figure 6A, in which patients are clustered in the two groups in order of age, almost all the miRNAs of PP patients show levels close to or lower than average, with six miRNAs (miR-31-3p, miR-126-3p, miR-133a-3p, miR-145-5p, miR-301a-3p and miR-532-5p) not detected in all five patients, and various miRNAs detected in only one or two patients. The high variability among individuals did not appear to correlate with their age (Figure 6A). The analysis of the average level of each miRNA across the two patient groups failed to show significant differences (Figure 6B). However, despite the discrepancy between the number of patients in the two groups, miR-149-5p and miR-598-3p, showing similar levels in both males and females, were significantly upmodulated in PP patients.

**Figure 7:**
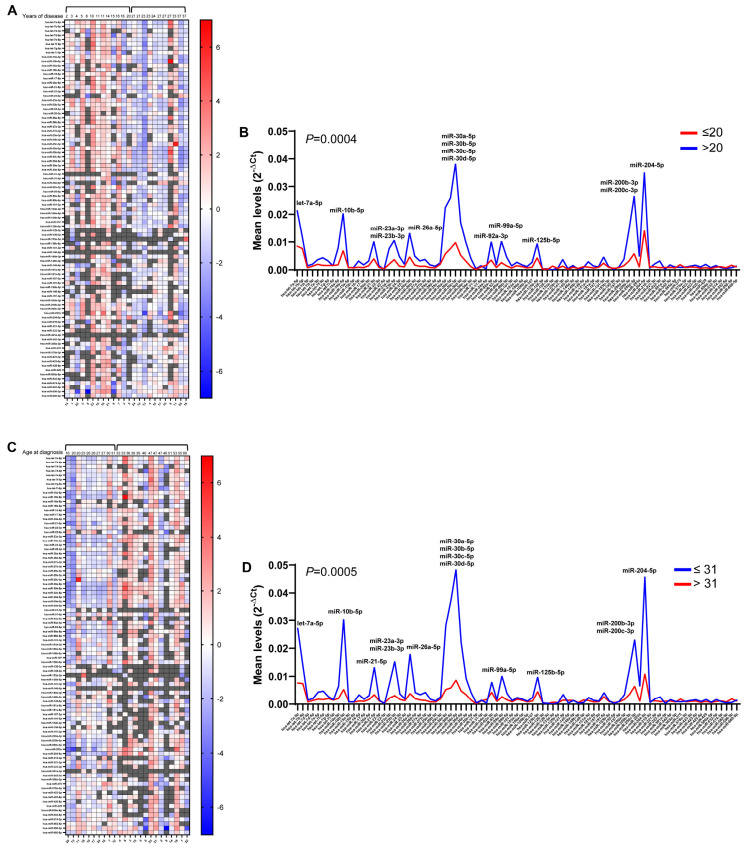
Correlation between levels of urinary exosomal miRNAs with age of disease. In (**A**,**C**), respectively, the correlation between years of illness or age at diagnosis and ΔCt values obtained by using the global mean expression of each miRNA as a reference are shown. Missing values are shown in gray. MiRNA intensities are displayed as colors ranging from blue (higher than average) to red (lower than average). In (**B**,**D**), respectively, line graphs represent the analysis of the mean expression of each miRNA in the two populations (≤20 and >20 or ≤31 and >31 years at diagnosis), expressed as 2^−ΔCt^, using a spike-in miRNA inside the plate as a reference. Significantly different miRNA levels are indicated.

We then evaluated the variability of urinary exosomal miRNAs throughout the duration of the disease, which showed an apparent direct correlation between years of disease and miRNA levels with respect to the average (Figure 7A). Stratification of patients based on the heat map data was performed, allowing us to identify over 20 years the cut-off between patients with miRNA levels lower or higher than average. The comparison of the average levels of each miRNA between the two groups allowed us to assess the significantly higher levels (*p* = 0.0004) of the examined miRNAs in the urinary exosomes of patients who had been diagnosed with MS for more than 20 years, with let-7a-5p, miR-10b-5p, miR-23a-3p, miR-23b-3p, miR-26a-5p, miR-30a-5p, miR-30b-5p, miR-30c-5p, miR-30d-5p, miR-92a-3p, miR-99a-5p, miR-125b-5p, miR-200b-3p, miR-200c-3p, and miR-204-5p being significantly upmodulated (Figure 7B).

When patients were clustered by age of insurgence of the pathology (Figure 7C), higher miRNA levels in samples from patients under 31 at the time of MS diagnosis was revealed (*p* = 0.0005), with upmodulation of most of the above identified miRNAs. In fact, apart from miR-92a-3p, the different levels of the above reported miRNAs between the two groups were amplified, and upmodulated miRNAs also included miR-21-5p (Figure 7D).

**Figure 8:**
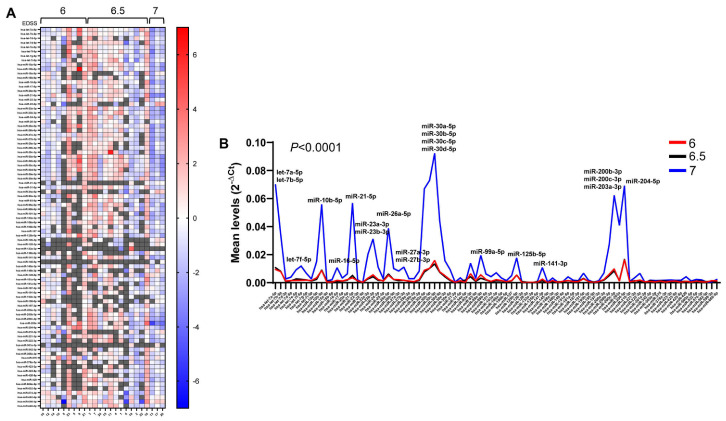
Correlation of urinary exosomal miRNAs with EDSS score. In (**A**), a heat map shows the ΔCt levels of urinary exosomal miRNAs in MS patients clustered by EDSS scores, obtained using the global mean expression of each miRNA as a reference. Missing values are shown in gray. MiRNA intensities are displayed as colors ranging from blue (higher than average) to red (lower than average). In (**B**), line graphs represent the analysis of the mean levels of each miRNA in the three groups, expressed as 2^−ΔCt^. Significantly different miRNA levels are indicated.

We further analyzed the relationship between urinary exosomal miRNAs and EDSS (Expanded Disability Status Scale) scores, a method widely used to quantify disability and monitor changes over time in people with multiple sclerosis [25]. Despite the patients we enrolled having similar EDSS scores, we revealed that patients with EDSS scores of 6 and 6.5 exhibit greater variability in miRNA expression than patients with scores of 7, corresponding to a greater disability (Figure 8A). A comparison of the average level of each miRNA in the three groups shows that patients with scores of 6 and 6.5 exhibit overlapping average levels, whilst patients with scores of 7 display an overall greater miRNA level (*p* < 0.0001), with some miRNAs differentially expressed (Figure 8B). Of note, the highly expressed miRNAs in patients with the highest EDSS scores are almost all the miRNAs upmodulated in patients who have had MS for more than 20 years, or patients with early insurgence. They also include let-7b-5p, let-7f-5p, miR-16-5p, miR-27a-3p, miR-27b-3p, miR-141-3p, and miR-203a-3p (Figure 8B).

**Figure 9:**
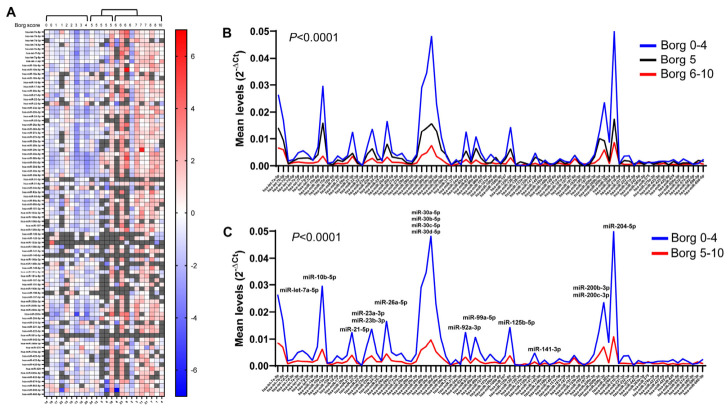
Correlation of urinary exosomal miRNAs with rate of perceived exertion. In (**A**), the correlation between Borg score and ΔCt values obtained using the global mean expression of each miRNA as a reference is shown. MiRNA intensities are displayed as colors ranging from blue to red. Missing values are shown in gray. In (**B**,**C**), line graphs represent the analysis of the mean expression of each miRNA in the patient groups, expressed as 2^−ΔCt^. Significantly different miRNA levels are indicated.

Finally, we correlated UE miRNAs with the Rate of Perceived Exertion (RPE) after a sub-maximal incremental test via treadmill to measure exercise tolerance. At the end of testing protocol [26], patients were asked to rate their perceived effort according to Borg’s 0–10 scale [27]. We revealed an inverse relationship between total UE miRNA levels and RPE (Figure 9A), with patients with a score of 5 showing highly variable miRNA levels. For this reason, we clustered the miRNA levels of patients into three groups, including 0–4, 5, and 6–10 RPE scores, showing that patients with lower score intervals had the highest miRNA levels (*p* < 0.0001) and a high number of differentially expressed miRNAs (Figure 9B). Despite revealing a progressive decrease in miRNAs, no significant differences were found between the two other groups. We then used score 4 as the cut-off, showing that patients with a RPE score ≤ 4 exhibit significantly higher average miRNA levels (*p* < 0.0001), with several miRNAs differentially expressed. Differentially expressed miRNAs included let-7a-5p, miR-10b-5p, miR-21-5p, miR-23a-3p, miR-23b-3p, miR-26a-5p, miR-30a-5p, miR-30b-5p, miR-30c-5p, miR-30d-5p, miR-92a-3p, miR-99a-5p, miR-125b-5p, miR-141-3p, miR-200b-3p, miR-200c-3p, and miR-204-5p (Figure 9C).

**Figure 10:**
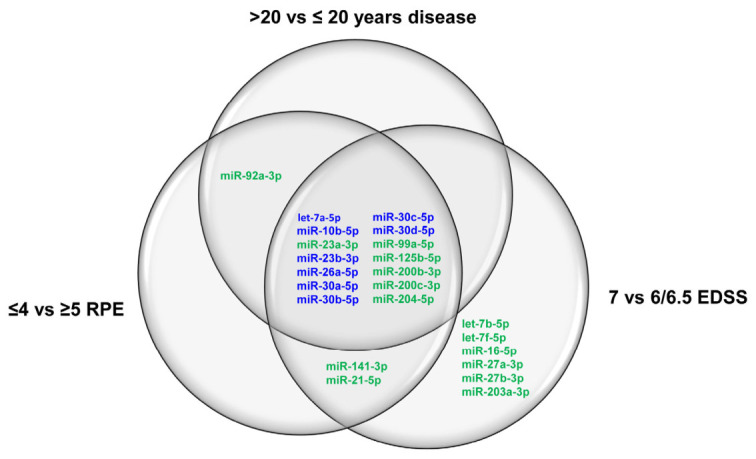
Venn diagram reporting the differentially expressed miRNAs among MS patient subgroups categorized by years of disease, EDSS score, and RPE using the Borg scale. The diagram highlights commonly upmodulated miRNAs across distinct categories. MiRNAs unique to a distinct group are shown within non-overlapping regions, while shared miRNAs are in intersecting areas. In blue are indicated upregulated miRNAs in female patients younger than 55 years.

Overall, the results obtained reveal a panel of 14 urinary exosomal miRNAs that characterize patients with more than 20 years of disease and a relatively high disability score but with a reduced perceived exertion (Figure 10), suggesting that miRNAs conveyed by urinary exosomes could furnish information to better stratify MS patients, with the aim of providing a more personalized therapeutic approach to care.

## 3. Discussion

Due to their stability, miRNAs encapsulated within extracellular vesicles, particularly exosomes, are promising biomarkers for a wide range of pathological conditions [28]. Exosomes can cross biological barriers, including the blood–brain barrier, making exosomal miRNAs helpful for also studying diseases of the central nervous system [13]. Due to the intrinsic fragility of patients with neurodegenerative diseases, urine is an appealing source of biomarkers, and altered levels of urinary exosomal miRNAs have been reported in animal models of Alzheimer’s disease (AD), reflecting their potential as markers of neuroinflammation and synaptic dysfunction [29]. Since studies in multiple sclerosis have focused on extracellular vesicles from serum and CSF [15,16,17], this investigation was aimed at exploring the potential of urinary exosomal miRNAs as non-invasive biomarkers for monitoring this central nervous disease. We evaluated a panel of 87 miRNAs in exosomes purified from the urine of 24 patients with progressive MS and severe disability, 17 of which were females, reflecting the correlation of MS with gender [2]. We selected a panel of urinary exosomal miRNAs variously correlated with neurogenesis, neuronal activity, and/or alterations of the cardiovascular system based on the progressive decline of cardiorespiratory functions associated with the high prevalence of motor dysfunction and gait disability in people with MS [30,31]. Although at highly variable levels between patients, 68 of the 87 examined miRNAs were found in at least two-thirds of the sample, and 11/87 miRNAs, including seven of the miRNAs showing the highest levels in our samples, were found in the urinary exosomes of all enrolled MS patients. This first evidence is in line with NGS data reporting the most abundant miRNAs in UEs [32,33], showing that the selected miRNA panel was evaluable in our UE samples.

As specific urinary exosomal miRNAs were reported to differ quantitatively between males and females, depending on hormonal regulation [34], and considering the prevalence of female patients in our MS cohort, we explored the relationship between the selected panel of urinary exosomal miRNAs with biological gender. As in healthy people [34], we revealed a significantly lower overall miRNA level in UEs from males than from females, where we identified a strong correlation with age. In fact, female patients younger than 55 years showed significantly higher levels of eight miRNAs (let-7a-5p, miR-10b-5p, miR-23b-3p, 26a-5p, miR-30a-5p, miR-30b-5p, miR-30c-5p, and miR-30d-5p) than older patients, in which levels were similar to those of males. These upmodulated miRNAs are strongly correlated with female hormones, being regulated by estrogen and/or involved in hormone-dependent pathways [35,36]. The low levels of these miRNAs that we revealed in the UEs from female patients aged between 55 and 60 could be related to the decline in estrogen that accompanies menopause and post-menopause, and their increase in subsequent years may be due to hormone replacement or steroid therapies. If, on one hand, this evidence confirms the expected relationship between urinary exosomal cargo and gender-related miRNA profiles [34], on the other hand, it allows us to identify miRNAs upregulated in younger females and that are strongly implicated in MS and/or cardiovascular diseases. They include let-7a-5p, found in plasma exosomes from MS patients, and correlated with progression and response to treatments [17,37], and miR-10b-5p, reported to be upregulated in animal MS models [38], in brains from patients with Huntington’s disease (HD), and in neuronal models of ALS (amyotrophic lateral sclerosis) [39], suggesting its general role in neurodegeneration. Upmodulated miRNAs in younger women also comprise miR-23b-3p, implicated in immune regulation and neuroinflammation, protection against demyelination [40], and involved in vascular remodeling and endothelial cell functions [41], whose levels in the plasma and CSF of MS patients were correlated with progression and response to DMF [37,42]. The serum and CSF levels of miR-26a-5p, regulating autophagy and NLRP3 inflammasome and mediating cardiac hypertrophy and myocardial infarction damage [43], were reported as biomarkers of MS [44]. Important relationships between miR-30 members that we found upmodulated in female patients younger than 55, and MS, were described. Concerning miR-30a-5p, elevated levels were found in serum exosomes, allowing us to discriminate between RRMS and PMS [15]. High serum exosome levels of miR-30b-5p were associated with MS activity and brain atrophy [15,45], while its decrease in erythrocytes and plasma EV was identified as a biomarker of RRMS and response to treatment [46,47]. On the other hand, while miR-30a-5p acts as a prognostic biomarker for ventricular dysfunctions, miR-30b-5p promotes myocardial cell apoptosis after infarction and regulates vascular smooth muscle cell differentiation [48]. This evidence holds particular interest considering that estrogen influences MS risk and may help explain why MS is more common in women [49]. Conversely, estrogen seems to play a protective role in MS, reducing inflammation and supporting nerve health [50,51], suggesting the need for a more in-depth analysis of estrogen-related miRNAs and the possibility that fluid exosomes, including urinary exosomes, could provide information on specific clinical aspects of MS in female patients. Of the miR-30 family, miR-30c-5p was also reported to increase in the CSF of MS patients, without significant clinical correlations [42].

We then tried to assess whether, in addition to the gender and age of MS patients, urinary exosomal miRNAs correlate with clinical parameters of the pathology. Although all our patients presented with progressive MS, and PPMS is clinically indistinguishable from SPMS [2,4], we revealed that the UEs of all PPMS patients lacked three miRNAs, which, at variance, were displayed by at least half of patients with SPMS. They include miR-31-3p, which is associated with autoimmunity and is downregulated in the peripheral blood mononuclear cells (PBMCs) of MS patients, potentially contributing to inflammatory responses [52]. In addition, the absence of miR-31 reduced the severity of a murine model of MS [53]. The miRNAs absent in UEs of our PPMS patients also included miR-126-3p, involved in endothelial function and angiogenesis [54], and dysregulated in the serum and PMBCs of MS patients, in which it correlates with disability progression and response to treatments [37,52,55]. A decrease in miR-126-3p can also contribute to blood–brain barrier dysfunction and neuroinflammation, both strongly correlated with disease activity and progression [55]. Finally, the serum exosomal levels of miR-532-5p, absent in the UEs of our PPMS patients, was reported to significantly decrease during RRMS relapses, and can be used as part of signatures that distinguish disease status [56]. Interestingly, in addition to the specific lack of three miRNAs, the UEs of our PP patients showed a significant upmodulation in miR-149-5p and miR-598-3p. Concerning miR-149-5p, it has a role in cancer suppression, inflammation and vascular biology [57,58,59], and was shown in increased levels in the gray matter of MS patients [60], where decreased following treatment with IFN-β [61]. At variance, miR-598-3p was identified in the CSF from AD patients [13] and in erythrocytes from RRMS patients [46], but its functional role remains unclear. Despite the discrepancy between the number of patients with PPMS (*n* = 5) and SPMS (*n* = 19) in our cohort, this second evidence suggests that urine could be a source of exosomes that better characterize the form of progressive MS that manifests de novo, without a preceding relapsing-remitting phase, allowing clinicians to anticipate PPMS diagnosis.

Since MS is a lifelong disease, with a very different progression timing among patients, we tried to correlate levels of urinary exosomal miRNAs with disease duration, revealing a direct relationship between patients affected by MS for more than 20 years and significantly higher levels of miRNAs in their UEs. Some of these (let-7a-5p, miR-10b-5p, miR-23b-3p, miR-26a-5p, miR-30a-5p and miR-30b-5p), despite their known relationship with MS, were upmodulated in female patients younger than 55, which constituted the majority of this group (6/8). On the other hand, in our cohort, seven miRNAs (miR-23a-3p, miR-92a-3p, miR-99a-5p, mir-125b-3p, miR-200b-3p, miR-200c-3p, and miR-204-5p) were specifically correlated with the duration of the pathology. They include miR-23a-3p, which was detected in the blood of MS patients and shows heterogeneous directionality depending on compartment (serum vs. plasma vs. exosomes), cohort, and method. Recent studies revealed reduced miR-23a-3p levels in relapsing MS or in people with brain atrophy [17]. On the other hand, miR-23a-3p participates in oligodendrocyte differentiation and increases within active and chronic MS lesions, particularly in RRMS [15,37,62,63]. MiR-92a-3p, whose UE levels were directly correlated with the duration of the pathology in our cohort, is part of the miR-17/92 cluster and linked to Th17 differentiation and inflammatory signaling often discussed in MS pathogenesis [44]. MiR-92a-3p was reported to increase in the CSF of MS patients and its circulating levels were correlated with white matter lesions and response to treatments [17,42,64]. Concerning miR-99a-5p, correlated with immune signaling, it was found to be upmodulated in the CD4+ T cells of MS patients [63]. Members of the miR-200 family correlated with disease duration seem to be implicated in MS pathogenesis. Possibly contributing to blood–brain barrier dysfunction, miR-200b-3p may influence immune cell trafficking into the CNS [65]. MiR-200c-3p and miR-200b show overlapping functions and were directly associated with MS in animal models [38]. However, miR-200c-3p was downregulated whereas miR-200b-3p was upregulated, suggesting differential regulation of miR-200 family members and a potential role in neuroinflammation and endothelial dysfunction relevant to MS pathology [66]. Also, miR-200c-3p seems to correlate with MS, as its serum levels were associated with disability progression [55]. Finally, miR-204-5p was reported in circulating exosomes in neurodegeneration and inflammation, without a clear correlation with MS [67]. With the exclusion of miR-92a-3p and with the addition of miR-21-5p, which is frequently enriched in exosomes from MS plasma and CSF [17,42,47], miRNAs correlated with the duration of the disease in our patient cohort were also upmodulated in the UEs from patients with MS insurgence before 31 years of age, confirming their relationship with the duration of the pathology, and once again suggesting that analysis of miRNAs in UEs could improve knowledge about the clinical conditions of MS patients.

We further analyzed the relationship between urinary exosomal miRNAs and EDSS score, used to quantify disability in people with multiple sclerosis [25]. Despite patients we enrolled having similar, relatively high EDSS scores, we revealed that individuals with the highest disability contain in their UEs a significantly greater level of 21 miRNAs. They included six miRNAs (let-7a-5p, miR-10b-5p, miR-26a-5p, miR-30b-5p, miR-30c-5p and miR-30d-5p) that also correlated with estrogen levels, and were hardly useful to monitor other events because all three patients of that group were females younger than 55 years. Nine more miRNAs (miR-21-5p, miR-23a-3p, miR-23b-3p, miR-30a-5p, miR-99a-5p, miR-125b-5p, miR-200b-3p, miR-200c-3p and miR-204-5p) were also upmodulated in patients who had suffered from MS for a longer duration, while six mRNAs (let-7b-5p, let-7f-5p, miR-16-5p, miR-27a-3p, miR-27b-3p, and miR-141-3p) were specifically upmodulated in patients with greater disability. Among them, let-7b-5p is variously involved in neurological disorders [68] and its level in the CSF, serum and plasma characterizes patients with progressive MS compared to those with RRMS, and high levels were linked to disability progression [37,69,70]. Reduced let-7b-5p in myeloid EV from MS patients was correlated with cognitive impairment [71]. In addition, in the non-progressive phase, the CSF levels of let-7b-5p show a negative correlation with both central and peripheral inflammation [70,71], and its level in the peripheral blood of patients with MS can be induced by IFN-β treatment [37]. The levels of let-7f-5p, linked to angiogenesis and vascular remodeling and altered in MS immune cells [72], increased in the erythrocytes from MS patients, without clear clinical correlations [46]. MiR-16-5p is upregulated in the CSF of MS patients, particularly in patients with less effective treatments and/or higher inflammatory disease, where it has been linked to apoptosis and immune regulation [42]. MiR-16-5p also plays a key role in cardiovascular pathophysiology, including cardiac remodeling, ischemia–reperfusion injury, and the regulation of vascular inflammation [73], highlighting its potential as a biomarker and therapeutic target. Concerning miR-27a-3p, its circulating level emerged as part of biomarker signatures distinguishing RRMS from progressive MS [74]. Its levels in immune cells were correlated with progression and response to treatments [37,52,63]. Furthermore, miR-27a-3p exerts regulatory effects on endothelial cells, promoting the expression of tight junction proteins and contributing to blood–brain barrier integrity [75]. With respect to miR-27b-3p, also in MS patients it participates in immune regulation and influences vascular inflammation and atherogenesis [76,77]. Together with miR-23a-3p and miR-23b-3p, circulating levels of miR-27a-3p and miR-27b-3p have been reported as predictive biomarkers for MS progression and response to dimethyl fumarate (DMF) treatment [37,42]. These miRNAs not only correlate with changes in disability, as assessed by the EDSS Scale, but also distinguish good responders from poor responders to DMF therapy, highlighting their potential utility for the personalized monitoring of treatments [37]. Lastly, blood miR-141-3p is upmodulated in PBMCs from relapsed RRMS patients [78], and plays roles in endothelial dysfunction in the brain and vascular stress responses [79,80].

We finally tried to correlate the levels of urinary exosomal miRNAs with the perceived exertions by patients at the end of an incremental treadmill test. Interestingly, we observed an inverse relationship between the overall levels of miRNAs in UEs and the RPE, with miRNAs significantly upmodulated in patients with low scores overlapping those in samples from patients with a longer disease duration and/or with a high EDSS score.

This analysis highlights how the majority of the urinary exosomal miRNAs that we were able to correlate with the various characteristics of our patient cohort were already described as deregulated in MS in comparison with healthy subjects (Table 1). Furthermore, despite differences in the biological sources across studies (blood, CSF, exosomes from both fluids), most of them have been correlated with the clinical characteristics of MS patients (Table 1), confirming the potential of urinary exosomes as non-invasive sources of biomarkers for monitoring MS. Unfortunately, some miRNAs considered promising markers for monitoring neuroinflammation, progression and/or response to treatment (such as miR-155, miR-92, miR-223) [17,81,82,83,84,85], were not examined as they were not included in the panel we selected to specifically detect miRNAs present in urinary exosomes that potentially correlate with MS and/or cardiovascular disorders, which contribute to motor dysfunction in people with MS [30,31].

## 4. Conclusions

Our study provides the first comprehensive characterization of urinary exosomal miRNAs in patients with progressive MS, demonstrating that these miRNAs are detectable, stable, and reflect both disease-specific and systemic alterations. We identified a panel of 14 upmodulated miRNAs consistently present across patients and with established roles in immune regulation, neuroinflammation, endothelial function, and cardiovascular signaling, associated with disease activity, disability progression, or long disease duration, answering the question of whether urinary exosomal miRNAs are potential non-invasive biomarkers for monitoring MS pathophysiology. Furthermore, the inverse correlation between miRNA abundance in urinary exosomes and perceived exertion suggests that they could also reflect functional or systemic disease severity impacting the function of the musculoskeletal system.

Nonetheless, this study has some limitations, the main one of which is the relatively low number of enrolled MS patients, all with progressive pathology, but with an uneven distribution between PPMS and SPMS. Also, the narrow range of disability scores in the cohort limited robust assessment of associations with clinical severity, and the absence of healthy controls prevented us from definitively attributing observed miRNA changes specifically to MS. Collectively, these limitations underscore the need for larger, longitudinal studies with appropriate controls and functional analyses to validate the prognostic potential of urinary exosomal miRNAs in MS.

Taken together, our findings suggest urinary exosomal miRNA cargo as a promising, non-invasive source of biomarkers that capture both CNS-specific and systemic aspects of MS, providing a foundation for future studies exploring their diagnostic, prognostic, and therapeutic utility and possible responses to rehabilitation treatments.

## 5. Materials and Methods

All reagents were obtained from Merck KGaA (Darmstadt, Germany) unless otherwise indicated.

### 5.1. Patients

The study was conducted on the urine exosomes from 24 patients (seven males and 17 females) with primary (*n* = 5) or secondary (*n* = 19) progressive MS, recruited at the “Outpatient Rehabilitation Clinic” at the University Hospital of Ferrara in the context of the PROGR-EX trial, a motor rehabilitation trial for studying people with multiple sclerosis and severe gait disability [86,87]. Patient age ranged from 41 to 64 years (mean 55 ± 6), the duration of pathology from 2 to 37 years (mean 18.2 ± 10.3), and EDSS scores ranged from 6 to 7. Patients were subjected to the evaluation of RPE according to Borg’s 0–10 scale [27] using a sub-maximal incremental test, adapted from Manfredini et al. [26], starting at 0.2 km/h without slope and progressing each minute by 0.2 km/h until a speed of 1 km/h was attained. The mean RPE of MS patients at the end of the testing protocol was 4.7 (±2.7).

All patients provided written informed consent, and the study was approved by the local Ethics Committee (Ethics Committee for the Central Emilia Wide Area—CE-AVEC, protocol code: EM417-2023_845/2019/Sper/AOUFe_EM1).

Patients were excluded if they had other neurological conditions, presented relapses or confounding factors during the study, or if they had received a botulinum toxin injection in the 3 months preceding the start of the study [86].

### 5.2. Purification and Characterization of Urinary Exosomes

Each of the 24 patients provided 20–50 mL of urine that was collected in the morning by staff at the University Hospital of Ferrara. The samples were stored at 4 °C after the addition of a cocktail of protease inhibitors (1 μg/mL aprotinin, 1 μg/mL leupeptin, 1 mM PMSF) and phosphatase inhibitors (1 mM Na_3_VO_4_), in order to avoid protein degradation and preserve the integrity of the exosomes. The purification process of the UEs was performed following well-known procedures. We started with centrifugation of the urine at 2000× *g* for 20 min at 4 °C to eliminate cells, cellular debris, bacteria and apoptotic bodies, followed by a centrifugation of the previously obtained supernatant at 16,000× *g* at 4 °C for 20 min to remove smaller cellular debris and larger vesicles [88]. The supernatant was then subjected to ultracentrifugation at 100,000× *g* for 2 h at 4 °C. The obtained pellet, corresponding to the exosomal fraction, was resuspended in 200 μL of PBS 1X and stored at −80 °C [89].

A transmission electron microscope was used to evaluate the fraction corresponding to exosomes. A negative staining procedure was used, following a previously described procedure [90]. The protocol required the deposition of 5 µL of sample on a copper grid coated with a graphite film of formvar, a thermoplastic resin that serves as a support for the sample, and subsequent incubation for 20 min. The samples were then fixed for 20 min with a phosphate-buffered solution containing 1% glutaraldehyde and 2% PFA. After six washes in water, slides were stained with a solution containing 0.5% uranyl acetate. The grid was observed with the Talos L120C G2 TEM (Thermo Fisher Scientific, Waltham, MA, USA) equipped with a 120 kV LaB6 thermionic source.

Purified extracellular vesicles were investigated for positivity against the exosomal markers CD63 [21] and Alix. The exosome pellet obtained from 100 mL of urine was lysed with RIPA Buffer added with protease inhibitor, boiled for 10 min with 4× loading buffer, separated on 8.5% polyacrylamide denaturing gel, and blotted onto a nitrocellulose membrane (GE Healthcare Life Science, Little Chalfont, UK). The membrane was probed overnight at 4 °C with an antibody directed against CD63 (#SAB4301607, Merck KGaA) and against Alix (#CQA2808, Cohesion Biosciences, London, UK), then incubated with a Goat anti-Rabbit IgG secondary antibody (Merck KGaA), followed by detection of immunocomplexes by using a WESTAR NOVA 2.0 kit (Cyanagen, Bologna, Italy). The chemiluminescence-derived bands were captured with an ImageQuantTM LAS 4000 imager (GE Healthcare Life Science) and quantified with Image Quant TL software v7.0 (GE Healthcare Life Science), as previously reported [91].

### 5.3. RT-qPCR Analysis of Urinary Exosomal miRNAs

Total RNA was extracted from urinary exosomes using the miRNeasy Micro Kit (Qiagen SpA, Milan, Italy), as previously described [92], with slight modifications as suggested by Levstek et al. [93]. Specifically, the exosomes were lysed with 800 μL of QIAzol, previously added with UniSp2, UniSp4, and UniSp5 spike-ins to control RNAs (miRCURY LNA RNA Spike-in kit, Qiagen SpA). Subsequently, 150 μL of chloroform was added to separate the aqueous phase containing the RNA from the organic phase containing the proteins. The procedure was completed following the manufacturer’s instructions.

The quality and quantity of extracted RNA were assessed using an Agilent Technologies Cary 60 UV–Vis spectrophotometer (Agilent Technologies, Santa Clara, CA, USA).

The obtained RNA was subsequently reverse transcribed to cDNA using the miRCURY LNA RT kit (Qiagen SpA), following the manufacturer’s instructions. Briefly, the reverse transcription mix was composed of 5× miRCURY RT Reaction Buffer, 10× miRCURY RT Enzyme Mix, UniSp6 and cel-miR-39-3p spike-in mix (miRCURY LNA RNA Spike-in kit, Qiagen SpA), 10 ng of RNA, and RNase-free water, in a final volume of 10 μL. Samples were loaded onto the thermal cycler and the following reverse transcription program was run: the first step at 42 °C for 60 min, the second step at 95 °C for 5 min, and finally, a storage step at 4 °C. The obtained cDNA was subsequently used for quantitative PCR.

For qRT-PCR, a mixture of 2× miRCURY SYBR Green Master Mix, ROX Reference Dye (Qiagen SpA), cDNA, and RNase-free water was prepared. A total of 10 μL of this mixture was dispensed into a 96-well plate (339325, YAHS-123Y—Human Urine Exosome Focus miRCURY LNA miRNA Focus PCR Panel from Qiagen) containing the specific primers for each miRNA under examination and for the RNA spike-ins introduced during extraction and reverse transcription. The plate was finally loaded onto a QuantStudio 3 thermocycler (Thermo Fisher Scientific, Waltham, MA, USA), and the following program was run: activation phase at 95 °C for 2 min, 40 cycles of denaturation (95 °C for 10 s), and annealing/extension (56 °C for 60 s). At the end of the reaction, a melting curve analysis was performed. Expression levels were normalized using the UniSp2 RNA spike-in and/or the global mean of expressed miRNAs, through the 2^−ΔCt^ or the 2^−ΔΔCt^ methods.

### 5.4. Statistical Analysis

Heat maps, graphs, and statistical analyses were produced using the GraphPad Prism 6.0 software. Statistical significance between independent study groups was assessed using Student’s *t*-test or one-way ANOVA, as appropriate, and *p* values < 0.05 were considered statistically significant.

## Figures and Tables

**Table 1:** Differentially expressed miRNAs in multiple sclerosis.

miRNA	Source	MS vs. HC	Clinical Correlation	Ref.
let-7a-5p	Plasma EVs	No HC	Response to treatment	[17]
	Plasma	Similar	Progression, response to DMF	[37]
let-7b-5p	Plasma	Similar	Progression, response to DMF	[37]
	CSF	Variable with subtype	PMS vs. RRMS	[69]
	CSF, serum	No HC	PPMS characterization	[70]
	Plasma myeloid EVs	Decreased	Cognitive impairment	[71]
let-7f-5p	Erythrocytes	Increased	─	[46]
	CD4+ T cells	Reduced	─	[72]
miR-16-5p	CSF	Increased	─	[42]
miR-21-5p	Plasma EVs	No HC	Response to treatment	[17]
	CSF	Increased	─	[42]
	Plasma EVs	Increased	Response to treatment	[47]
miR-23a-3p	Serum exosomes	Increased	Distinguish RRMS from PMS	[15]
	Plasma EVs	No HC	Brain atrophy, relapses	[17]
	Plasma	Similar	Progression, response to DMF	[37]
	Plasma	Increased	Active and chronic lesions	[62]
	CD4+ T cells	Increased	─	[63]
miR-23b-3p	Plasma	Similar	Progression, response to DMF	[37]
	CSF	Decreased	─	[42]
miR-26a-5p	CSF, serum	No HC	MS characterization	[70]
miR-27a-3p	Plasma	Similar	Progression, response to DMF	[37]
	PMBCs	Increased	Response to treatment	[52]
	CD4+ T cells	Increased	MS characterization	[63]
	CD4+ T cells	Similar	Distinguishing RRMS from PMS	[74]
miR-27b-3p	Plasma	Similar	Progression, response to DMF	[37]
	CSF	Decreased	─	[42]
miR-30a-5p	Serum exosomes	Increased	Distinguish RRMS from PMS	[15]
miR-30b-5p	Serum exosomes	Increased	Distinguish RRMS from PMS	[15]
	Serum	No HC	Brain atrophy	[45]
	Erythrocytes	Decreased	RRMS biomarker	[46]
	Plasma EVs	Decreased	Response to treatment	[47]
miR-30c-5p	CSF	Increased	─	[42]
miR-31-3p	PBMCs	Reduced	Response to treatment	[52]
miR-92a-3p	Plasma EVs	No HC	Response to treatment	[17]
	Plasma	Similar	─	[37]
	CSF	Increased	Response to treatment	[42]
	Serum	No HC	White matter lesion	[64]
miR-99a-5p	CD4+ T cells	Increased	─	[63]
miR-125b-5p	Plasma EV	No HC	Brain atrophy, response to treatment	[17]
	CD4+ T cells	Increased	─	[63]
miR-126-3p	Plasma	Similar	Progression, response to DMF	[37]
	PMBCs	Increased	Response to treatment	[52]
	Serum	No HC	Progression disability	[55]
miR-141-3p	PMBCs	Increased	Progression	[78]
miR-149-5p	Gray matter, serum	Increased	Brain atrophy	[60]
miR-200c-3p	Serum	No HC	Progression disability	[55]
miR-203a-3p	PMBCs	Increased	Progression	[78]
miR-532-5p	Serum exosomes	Decreased	RRMS relapses	[56]

HC: healthy control; MS: multiple sclerosis; PMS: progressive multiple sclerosis; RRMS: relapsing-remitting multiple sclerosis; DMF: dimethyl fumarate; EVs: extracellular vesicles.

## Data Availability

All data are contained within the article.
